# Digital scanning is more accurate than using a periodontal probe to measure the keratinized tissue width

**DOI:** 10.1038/s41598-020-60291-0

**Published:** 2020-02-28

**Authors:** Jung-Seok Lee, Yoon-Sun Jeon, Franz-Josef Strauss, Hoi-In Jung, Reinhard Gruber

**Affiliations:** 1 Department of Periodontology, Research Institute for Periodontal Regeneration, College of Dentistry, Yonsei University, Seoul, Republic of Korea; 2 Clinic of Reconstructive Dentistry, Center of Dental Medicine, University of Zurich, Zurich, Switzerland; 3 Department of Oral Biology, School of Dentistry, Medical University of Vienna, Vienna, Austria; 4 Department of Conservative Dentistry, School of Dentistry, University of Chile, Santiago, Chile; 5 Department of Preventive Dentistry & Public Oral Health, College of Dentistry, Yonsei University, Seoul, Republic of Korea

**Keywords:** Oral anatomy, Periodontitis

## Abstract

This study aimed to compare the accuracy and reliability of digital versus conventional clinical measurements of the width of keratinized tissue. To this end, the keratinized tissue width was measured at 110 tooth sites in 5 pig jaws. The measurements were made at each site using three-dimensional (3D) scanned images and a periodontal probe. The actual keratinized tissue width was subsequently measured on histologic slides prepared from the same sites, and differences between the histologic slides and the digital and clinical measurements were analyzed to determine their accuracy in two measurement rounds. Furthermore, intrarater and interrater reliabilities were evaluated using the intraclass correlation coefficient (ICC). Here we show that the mean differences (and lower/upper limits of agreement) between the histologic and the digital/clinical measurements were 0.10 mm (−1.34/1.54 mm) and 1.11 mm (−0.69/2.92 mm), respectively, in the first round of measurements (*p* < 0.01), and 0.04 mm (−1.52/1.59 mm) and 1.05 mm (−0.37/2.48 mm) in the second round of measurements (*p* < 0.01). Moreover, we found that the intrarater reliability was higher for the digital measurements (ICC = 0.97, confidence interval [CI] = 0.96–0.97) than for the clinical measurements (ICC = 0.87, CI = 0.86–0.89; *p* < 0.01). Taken together, our results demonstrate that digital measurements of the keratinized tissue width using 3D scanned images can replace conventional clinical measurements using a periodontal probe since they are more accurate and reliable.

## Introduction

The keratinized mucosa is considered an indispensable structure for the stability of the peri-implant tissues. This opinion is supported by findings that augmenting keratinized tissues improves peri-implant health, reduces mucosal inflammation, and results in less marginal bone loss around implants^1^. The dimensions of the keratinized mucosa are thus clinical parameters that are evaluated to predict the stability of the peri-implant health^2^. The keratinized mucosa can be distinguished from the non-keratinized mucosa by the coral pink color of its outermost surface; this is a consequence of light not being able to penetrate the keratinized epithelial cell multilayer, as revealed by histologic analysis^3^.

The dimensions of the keratinized mucosa have traditionally been clinically determined using a periodontal probe^4^. However, this method has limitations and is prone to inherent errors, including variations in placement and angulation along with rounding errors^5,6^. The rounding errors are particularly significant since they might substantially influence periodontal measurements in clinical and epidemiologic studies. Although these limitations can be reduced by using periodontal probes with close interval markings or individually customized stents, they are still susceptible to errors. One alternative might be to use digital technology, which is emerging as a novel potential option for overcoming these limitations. However, there is still insufficient evidence to support such analyses, and the accuracy and reliability of digital measurements have yet to be established.

The use of digital technology in dentistry has become tremendously popular, with its utilization expanding rapidly in various clinical and research fields^7,8^. Intraoral scanning can provide data on the shape of the outermost surfaces of teeth and dental implants, and recently has also been used to identify surrounding soft-tissue structures based on differences in color^9^. It is thus reasonable to suggest that the dimensions of the keratinized mucosa could be measured using intraoral scanning given the color difference between keratinized and nonkeratinized tissues. Considering the aforementioned limitations of periodontal probes and the utility of scanners in identifying tissue colors and contours, it can be hypothesized that digital scanners are useful for measuring the dimensions of the keratinized mucosa. Therefore, the aim of the present study was to compare the accuracy and reliability of digital measurements of the keratinized tissue width based on the three-dimensional (3D) scanned data with conventional clinical measurements.

## Results

### Histologic observations and measurements

**Figure 1:**
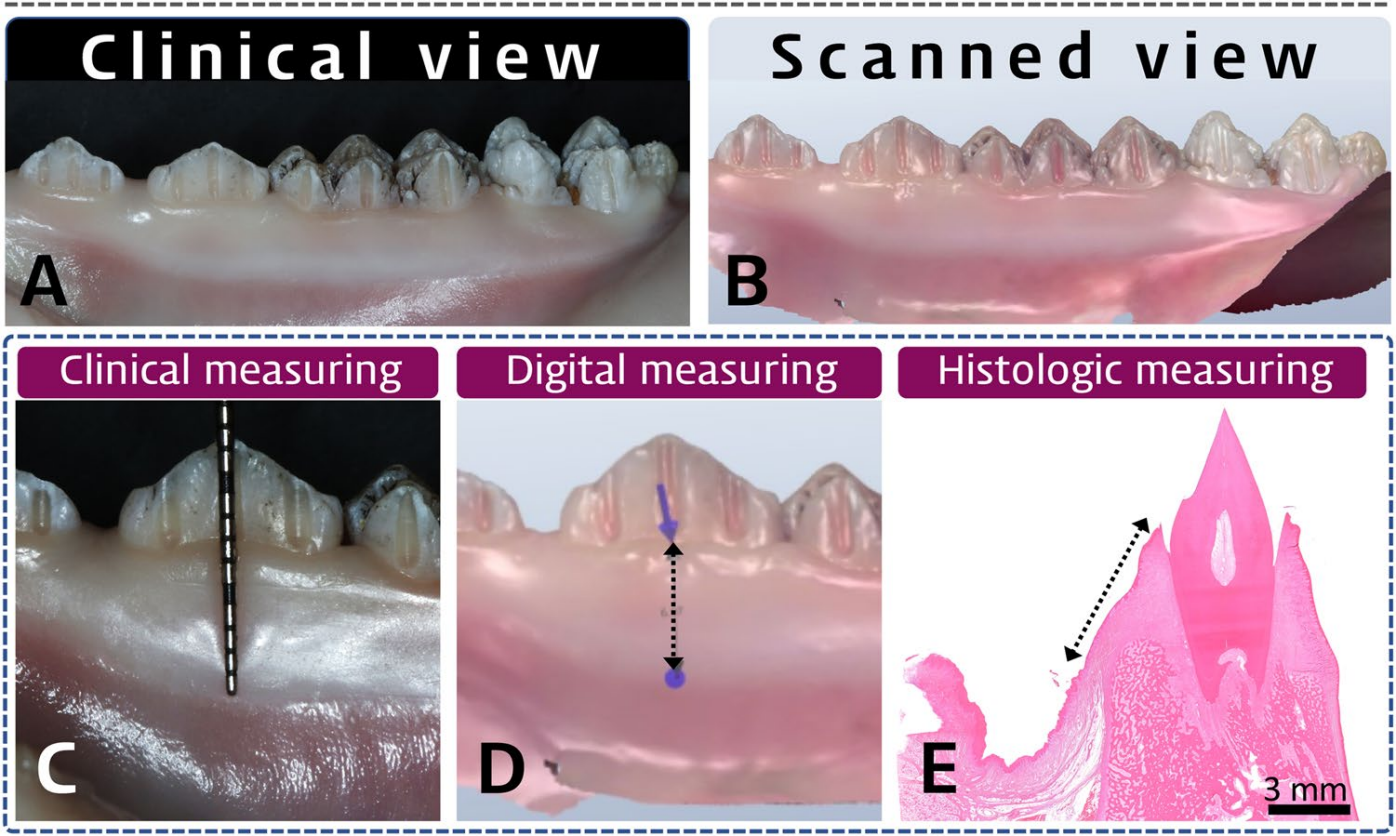
Representative clinical (**A**) and digitally scanned (**B**) views of the experimental pig-jaw model, and each measurement protocol: clinical (**C**), digital (**D**), and histologic (**E**) measurements. The three-dimensional scanned image shows teeth and the surrounding soft tissue, and the mucogingival junction is clearly distinguishable by the color differences. Every involved tooth has two or three prepared grooves on its crown, and was used as a reference for applying the instrument (**C**), guiding the measurements (**D**), and histologic sectioning (**E**). In both the clinical and digital measurements, the keratinized tissue width was measured based on its appearance and its difference in color from the adjacent mucosa. However, histologic measurements of keratinized tissue were based on the different histologic appearances of the layers of the epithelium and the connective tissue.

**Figure 2:**
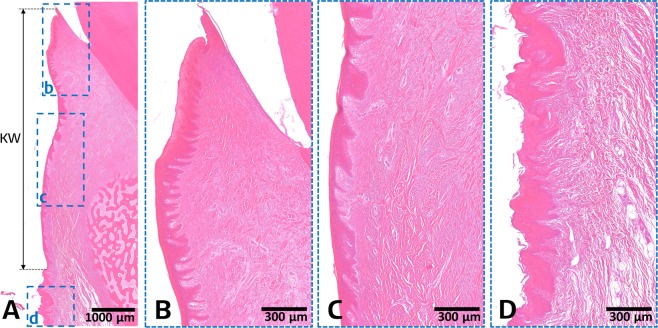
Representative histomicrographs of a buccolingual section at the experimental site: overview (**A**), gingival sulcus region (**B**), attached gingiva region (**C**), and mucosa region (**D**). The gingival sulcus and attached gingiva regions appear as keratinized, stratified squamous epithelial cell layers with numerous rete pegs and underlying dense connective tissue layer. In contrast, the mucosa region shows a nonkeratinized, thin epithelial layer and underlying loose connective tissue. Based on these specific histologic features, the mucogingival junction can be clearly distinguished and the keratinized tissue width (KW) can be measured.

We measured the width of keratinized gingiva using three different methods (clinical, digital, and histologic measuring) in the prepared pig jaw models (Fig. 1). To measure the actual dimensions of keratinized tissue histologically as a reference, three parts of gingiva and oral mucosa were identified based on their histologic appearances (Fig. 2): (1) in the gingival sulcus region (Fig. 2B), a single cell layer of sulcular epithelium freely contacted the coronal enamel surfaces and underlying dense connective tissue; (2) in the attached gingiva region (Fig. 2C) there were keratinized, stratified squamous epithelial cell layers with numerous rete pegs and underlying dense connective-tissue layer attached to the dental root and alveolar bone surfaces; and (3) the mucosa region (Fig. 2D) comprised a nonkeratinized, thin epithelial layer and underlying loose connective tissue. Thus, the mucogingival junction could be clearly distinguished based on these histologic differences, from which the histologic keratinized tissue width was measured to the most-coronal point of the free gingiva.

### Digital measurements of the keratinized tissue width are more accurate than conventional measurements

**Figure 3:**
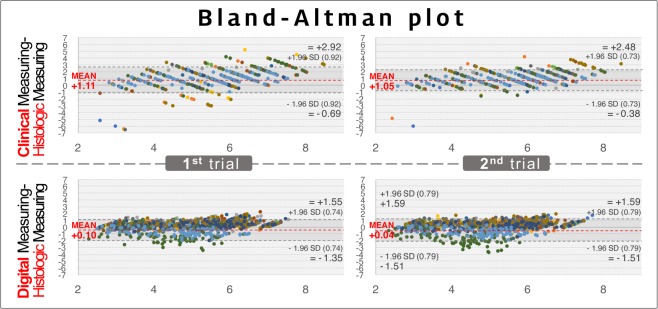
Bland-Altman plot showing the accuracy of the clinical and digital measurements in the first and the second round of measurements. In this plot, the *x*-axis indicates the average of the histologic measurements and either the clinical or digital measurements [(clinical or digital measurements + histologic measurements)/2], and the *y*-axis indicates the difference between the histologic measurements and either the clinical or digital measurements (clinical or digital measurements – histologic measurements). Based on the reference of the histologic measurements, the clinical measurements show a 1-mm overestimation in the first and second rounds of measurements, while both rounds of digital measurements demonstrate values similar to the histologic measurements. Dots of the same color indicate measurements made by the same examiner, and hence there are 12 colors for the 12 examiners.

**Figure 4:**
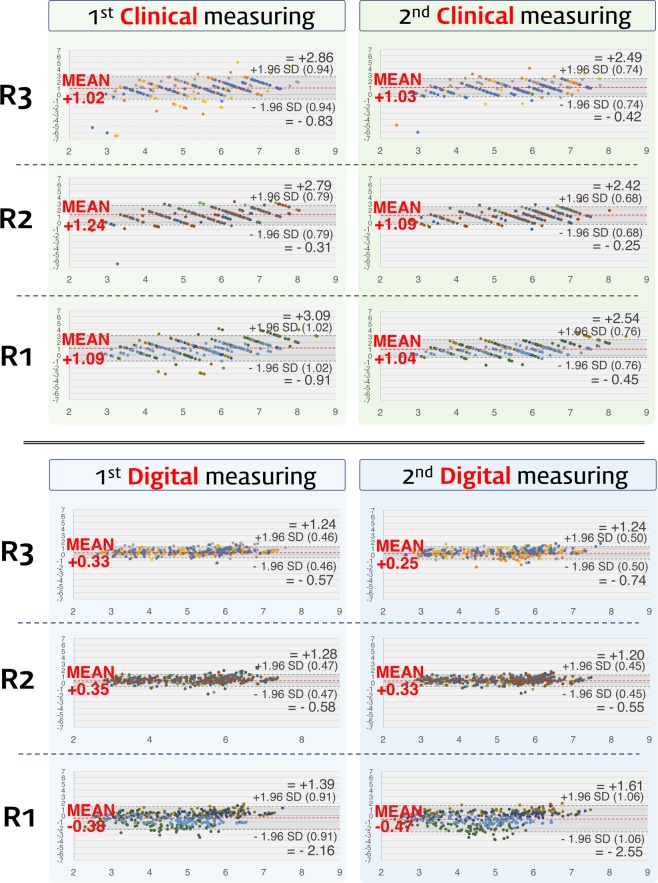
Bland-Altman plots for residents in each year. The accuracy patterns were similar for all of the residents, and subgroup analyses also showed the 1 mm of overestimation in the clinical measurements. R1, R2, and R3 are the first-, second-, and third-year residents, respectively.

We next calculated how much the clinical and digital measurements differed from the histologic keratinized tissue width. The differences were analyzed using Bland-Altman plots in the first and second rounds of measurements (Figs. 3 and 4). In both rounds the clinically measured values were concentrated at 1 mm larger than the histologic values, indicating a mean overestimation of 1 mm. In contrast, the differences between the digital measurements and histologic values were scattered around zero in both rounds, indicating a high accuracy. Although the clinical and digital measurements exhibited similar dispersion ranges, outliers beyond the upper and lower limits of the plots were significantly further from the average accuracy in the clinical measurements than in the digital measurements.

Based on the histologic measurements of the keratinized tissue width, statistical analyses further confirmed that the accuracy was significantly higher for the digital measurements than the clinical measurements in every examiner and in both rounds of measurements (*p* < 0.01; Table 1). The mean differences (and lower/upper limits of agreement) between the histologic and the digital/clinical measurements were 0.10 mm (−1.34/1.54 mm) and 1.11 mm (−0.69/2.92 mm), respectively, in the first round (*p* < 0.01), and 0.04 mm (−1.52/1.59 mm) and 1.05 mm (−0.37/2.48 mm) in the second round (*p* < 0.01). Together these results indicate that digital measurements of the keratinized tissue width are more accurate than conventional measurements.

**Table 1:** Comparison of the clinical and digital measurements by Bland-Altman analysis.

	Examiner	Clinical measurements minus histologic measurements			Digital measurements minus histologic measurements			*p*^**☨**^
		Bias^*^ (mm)	LLA (mm)	ULA (mm)	Bias^*^ (mm)	LLA (mm)	ULA (mm)	
First round	**Year 3**	1.02	−0.83	2.86	0.33	−0.57	1.24	<0.01
	**1**	1.19	−1.20	3.58	0.24	−0.57	1.04	<0.01
	**2**	1.05	−0.27	2.37	0.54	−0.38	1.47	<0.01
	**3**	0.82	−1.23	2.86	0.30	−0.56	1.17	<0.01
	**4**	1.01	−0.28	2.31	0.25	−0.64	1.14	<0.01
	**Year 2**	1.24	−0.31	2.79	0.35	−0.58	1.28	<0.01
	**5**	1.46	0.03	2.89	0.28	−0.61	1.18	<0.01
	**6**	0.97	−0.37	2.30	0.43	−0.58	1.45	<0.01
	**7**	1.16	−0.70	3.02	0.36	−0.56	1.29	<0.01
	**8**	1.36	0.04	2.68	0.31	−0.54	1.16	<0.01
	**Year 1**	1.09	−0.91	3.09	−0.38	−2.16	1.39	<0.01
	**9**	1.19	−1.52	3.90	0.39	−0.52	1.30	<0.01
	**10**	0.94	−0.32	2.21	0.16	−0.80	1.11	<0.01
	**11**	1.36	−0.85	3.57	−1.27	−3.01	0.47	<0.01
	**12**	0.86	−0.27	2.00	−0.81	−1.75	0.14	<0.01
	**Total**	1.11	−0.69	2.92	0.10	−1.34	1.54	<0.01
Second round	**Year 3**	1.03	−0.42	2.49	0.25	−0.74	1.24	<0.01
	**1**	1.47	0.01	2.93	0.04	−1.03	1.11	<0.01
	**2**	1.23	0.06	2.39	0.33	−0.61	1.26	<0.01
	**3**	0.73	−0.65	2.12	0.28	−0.61	1.18	<0.01
	**4**	0.69	−0.48	1.87	0.35	−0.57	1.27	<0.01
	**Year 2**	1.09	−0.25	2.42	0.33	−0.55	1.20	<0.01
	**5**	1.31	0.00	2.62	0.19	−0.60	0.97	<0.01
	**6**	0.97	−0.27	2.21	0.35	−0.55	1.25	<0.01
	**7**	0.83	−0.57	2.23	0.31	−0.58	1.20	<0.01
	**8**	1.24	0.09	2.40	0.46	−0.39	1.30	<0.01
	**Year 1**	1.04	−0.45	2.54	−0.47	−2.55	1.61	<0.01
	**9**	1.58	−0.01	3.16	0.52	−0.35	1.39	<0.01
	**10**	1.03	−0.17	2.22	0.03	−0.90	0.96	<0.01
	**11**	0.83	−0.75	2.42	−1.64	−3.44	0.16	<0.01
	**12**	0.74	−0.15	1.63	−0.78	−1.72	0.17	<0.01
	**Total**	1.05	−0.37	2.48	0.04	−1.52	1.59	<0.01

^*^Bias was estimated as the mean difference between the clinical/digital measurements and the histologic measurements.

LLA, lower limit of agreement (=mean – 1.96×standard deviation); ULA, upper limit of agreement (=mean + 1.96×standard deviation).

^**☨**^Paired *t*-test.

### Digital measurements of the keratinized tissue width are highly reliable

#### Intrarater reliability

To evaluate the reproducibility within each examiner, the intrarater reliability for the clinical and digital measurements was calculated using ICC values and confidence intervals (CIs) between the first and second rounds of measurements in all 12 examiners (Table 2). The average ICC values were 0.87 (CI = 0.86⎯0.89) and 0.97 (CI = 0.968⎯0.974) for the clinical and digital measurements, respectively. In the digital measurements, all examiners showed similarly high ICC values>0.95, except for one (ICC = 0.84). In contrast, the clinical measurements produced lower and a wider range of ICC values, with two examiners showing lower ICC values (<0.90) and another two having significantly lower ICC values (0.66 and 0.74). Statistical analysis based on the ICC values for individual examiners revealed that the intrarater reliability was significantly higher for digital measurements than for clinical measurements (*p* < 0.01).

**Table 2:** Intrarater reliability.

Examiner	Clinical measurements		Digital measurements^*^	
	ICC	CI	ICC	CI
1 (year 3)	0.66	0.51–0.77	0.95	0.93–0.97
2 (year 3)	0.90	0.86–0.93	0.97	0.96–0.98
3 (year 3)	0.84	0.76–0.89	0.97	0.96–0.98
4 (year 3)	0.95	0.93–0.97	0.98	0.97–0.99
5 (year 2)	0.93	0.90–0.95	0.98	0.97–0.99
6 (year 2)	0.94	0.91–0.96	0.97	0.95–0.98
7 (year 2)	0.91	0.87–0.94	0.99	0.98–0.99
8 (year 2)	0.91	0.86–0.94	0.98	0.96–0.98
9 (year 1)	0.74	0.61–0.82	0.96	0.94–0.97
10 (year 1)	0.94	0.92–0.96	0.97	0.96–0.98
11 (year 1)	0.81	0.73–0.87	0.84	0.77–0.89
12 (year 1)	0.96	0.94–0.97	0.997	0.996–0.998
Year 3	0.85	0.82–0.87	0.97	0.96–0.97
Year 2	0.92	0.91–0.94	0.98	0.97–0.98
Year 1	0.85	0.81–0.87	0.97	0.96–0.97
Total	0.87	0.86–0.89	0.97	0.968–0.974

^*^Digital measurements showed significantly higher intrarater reliability compared to clinical measurements (Wilcoxon signed-rank test, *p* < 0.01).

Intraclass correlation coefficients (ICCs) and confidence intervals (CIs) for comparisons between the first and second rounds of measurements for all 12 examiners.

#### Interrater reliability

We also evaluated the reproducibility between the 12 examiners (interrater reliability) based on the ICC values for all examiners or between residents in each year in each round of measurements; the results are presented Table 3. Overall, both the clinical and digital measurements produced high ICC values: 0.96 and 0.99, respectively, in the first round of measurements, and 0.98 and 0.99 in the second round of measurements. However, in the subgroup analysis of residents from each year, the first round of clinical measurements showed slightly lower ICC values compared to the second clinical measurements and the first digital measurements. Together these observations indicate that digital measurements of the keratinized tissue width are highly reliable.

**Table 3:** Interrater reliability. ICCs and CI for comparisons between all examiners and for residents in each year.

	Clinical measurements				Digital measurements			
	First round		Second round		First round		Second round	
	ICC	CI	ICC	CI	ICC	CI	ICC	CI
Year 3	0.89	0.85–0.92	0.95	0.93–0.96	0.99	0.98–0.99	0.98	0.98–0.99
Year 2	0.93	0.91–0.95	0.96	0.95–0.97	0.98	0.98–0.99	0.98	0.97–0.99
Year 1	0.87	0.82–0.90	0.93	0.91–0.95	0.93	0.90–0.95	0.92	0.89–0.94
Total	0.96	0.95–0.97	0.98	0.976–0.986	0.99	0.985–0.991	0.99	0.98–0.99

## Discussion

A precise method for measuring keratinized tissue is critical when assessing the success of soft-tissue augmentation techniques. However, most such measurements have been performed using periodontal probes, which have some inherent limitations. Here we took advantage of an intraoral scanner that can not only distinguish keratinized gingiva based on grades of coloration, but also measure the dimensions on the continuous-data basis, similar to the histologic appearance of the mucosa but non-invasively. The main finding of this study was that periodontal probes overestimated the width of the keratinized gingiva by around 1 mm, while the digital scanner closely reflected the findings obtained in the histologic analysis. Moreover, the digital measurements demonstrated significantly higher intrarater and interrater reliabilities compared to the traditional approach using a periodontal probe.

Error is an inherent part of physical measurements, and both the clinical and digital measurements made in this study also showed stochastic dispersion of the accuracy data (see the Bland-Altman plot in Figs. 3 and 4). Both the clinical and digital measurements were based on the clinical appearance of the outer surface of the mucosa, and possible errors in these two methods include the subjective bias of the examiners. However, clinical measurements exhibit further weak points of errors in both accuracy and reliability. A previous study that used a periodontal probe found differences within 1 mm when measuring the pocket depth^10^, which is consistent with the present study showing a 1-mm overestimation in clinical measurements made using the periodontal probe. Another previous study described visual approximation errors and positioning errors of the instrument when using a periodontal probe^11^, and these may also be aggravated by the inherent errors associated with this instrument.

The movability of the mucosa and the unclear boundary line of the mucogingival junction mean that it cannot be guaranteed that the tip of the periodontal probe is placed at the exact targeted position. Furthermore, errors might increase further if the examiner’s eyes are at a vertical level different from the reading point of the instrument. Although the present study used pig jaws, all examiners recorded the values in a standard way, in which the upper level of the examiner’s eye can increase the overestimation of values. Approximation errors can be another factor that reduces the accuracy of clinical measurements^12^. Each type of periodontal probe has its own graduated markings, and when a measurement lies between two marks, the clinician must round off to the nearest mark. This results in the clinically measured values being integers, and the present Bland-Altman plots showed that the clinical measurement data exhibited a specific linear pattern, rather than the data being randomly scattered (Figs. 3 and 4). The choice of rounding numbers up or down could only be made by the examiner, and so the clinical measurements could have included subjective bias. These two error sources could be synergistically integrated during conventional measurements, and might result in reduced accuracy and lower intrarater and interrater reliabilities.

Our findings on the accuracy and reliability of measuring keratinized gingiva dimension are directly related to clinical research, such as comparisons between various soft-tissue augmentation techniques. The errors that we found in the conventional measurements using a periodontal probe may overwhelm the range of mean differences in the outcomes between different techniques. Recent systematic reviews found mean differences between various techniques of less than 1 mm^13–17^, which makes measurements of the keratinized tissue critical when comparing different techniques. However, most of these results were based on values measured by reading a periodontal probe, and some studies have used measurement instruments with sparse markings over a wide interval (3 mm)^18,19^. Therefore, using digital measurements might be more robust in detecting differences between different surgical approaches and also less time-consuming.

This study was subject to some limitations. First, the data were based on a pig-jaw model that shows the same color pattern of mucosa seen clinically but with smaller dimensional variations of the keratinized mucosa. Second, the clinical application of the intraoral scanner for obtaining data on the soft tissue in the vestibular region can be hindered by surrounding anatomic structures such as the cheek or tongue. Moreover, the scanning process can be challenging in patients with restricted mouth opening or sudden movements. Hence, further clinical studies are needed to confirm the accuracy and reliability of digital measurements of the keratinized tissue width. Third, our study was based on a specific scanning algorithm, and so future studies should evaluate the accuracy and reliability of these digital measurements using other devices and protocols.

In summary, the present study has revealed that measuring the keratinized tissue width based on digital scanning is more accurate than conventional clinical measurements. Moreover, the digital measurements showed a higher intrarater reliability than clinical measurements, along with a high interrater reliability. These findings indicate that digital measurements of the keratinized tissue width on 3D scanned images can replace conventional clinical measurements using a periodontal probe and provide higher accuracy and reliability.

## Materials and Methods

### Preparation of the *in vitro* experimental model

This study obtained five pig jaws, and diamond burs were used to make vertical grooves on the dental crowns of two premolars and two molars in each jaw. In total, 550 grooves were placed in five jaw models (Fig. 1). These grooves were used as a reference to standardize the digital and clinical measurements of the keratinized mucosa, and in the histologic sections. The apicocoronal length of the grooves were measured by a vernier caliper as the reference length in calibrating histologic measurement. The pig jaws were then scanned with a digital oral scanner (TRIOS 3, 3Shape, Copenhagen, Denmark), and the obtained 3D images were saved.

### Study design

The accuracy of the measurements was determined by using the histologic length of the keratinized tissue as the true value of the keratinized tissue width. The differences between the histologically measured length and the digital/clinical measurements were analyzed. To evaluate the interrater and intrarater reliabilities, 12 resident dentists (first-, second-, and third-year residents; 4 from each year) from the Department of Periodontology at Yonsei University Dental Hospital performed clinical and digital measurements of the keratinized tissue width on the pig jaws and 3D scanned images, respectively. The measurements were repeated by all of the residents 1 hour after performing the first round of measurements.

### Measurements of the keratinized gingiva width

For calibration, one senior investigator (J.S.L.) explained how to perform the measurements to all 12 residents prior to the start of the assessment.

#### Clinical measurements

The examiners clinically measured the keratinized gingiva width using a UNC-15 periodontal probe (Hu-Friedy, Chicago, Illinois, USA). The periodontal probe was positioned in the groove made on the crown portion, and the length from the mucogingival junction to the marginal gingiva was measured. The 12 examiners made their first round of measurements on both alveolar ridges of the five pig jaws, and then performed the second round of measurements 1 hour later. All pig-jaw models were placed in a refrigerator after the first round in positions that were randomly rearranged in order to reduce bias in the repeated measurements.

#### Digital measurements

One week after the clinical measurements, the 12 examiners measured the distance from the mucogingival junction to the marginal gingiva on 3D scanned images of the pig jaws using computer software (OrthoAnalyzer, 3Shape). The measurements were made parallel with the grooves. One hour after the first round of digital measurements, the examiners performed a second round of measurements on randomly selected pig jaws.

#### Histologic measurements

All samples were fixed with 10% neutral buffered formalin for 1 week, and then demineralized for 28 days using Calci-Clear Rapid (National Diagnostics, Atlanta, GA, USA), which was changed every 48 hours. A specimen was cut at each measuring site along with the reference groove axis, and each histologic slide (4 μm) was prepared with paraffin embedding and hematoxylin-eosin staining. An expert (J.S.L.) measured the histologic distance from the marginal gingiva to the mucogingival junction, which could be clearly distinguished based on the histologic differences between the keratinized epithelium and dense connective tissue (Fig. 2). Each measurement was adjusted by the calibrated scale using the reference length of the groove in each site.

### Statistical analysis

Every measurement at every site was referred to as an individual unit. To evaluate the accuracy and agreement of the measurements, Bland-Altman plots were constructed for both the digital and clinical measurements using the histologic measurement as the reference value. The mean differences of the clinical and digital measurements from histologic measurement were assessed using the paired *t*-test. To test reliability, the intraclass correlation coefficient (ICC) was calculated for the correlations between the first and second rounds of the clinical/digital measurements (intrarater reliability), and between all examiners for the clinical/digital measurements (interrater reliability). Intrarater ICC values for the 12 examiners were evaluated for normality using the Shapiro-Wilk test, and compared using the Wilcoxon signed-rank test between clinical and digital measurements. The criterion for statistical significance was set at *p* < 0.05.

## Data Availability

The datasets generated during the current study are available from the corresponding author on reasonable request.
